# A compatibility evaluation between the physiologically based pharmacokinetic (PBPK) model and the compartmental PK model using the lumping method with real cases

**DOI:** 10.3389/fphar.2022.964049

**Published:** 2022-08-12

**Authors:** Hyo-jeong Ryu, Won-ho Kang, Taeheon Kim, Jae Kyoung Kim, Kwang-Hee Shin, Jung-woo Chae, Hwi-yeol Yun

**Affiliations:** ^1^ Department of Pharmacy, College of Pharmacy, Chungnam National University, Daejeon, South Korea; ^2^ Department of Mathematical Sciences, Korean Advanced Institute of Science and Technology, Daejeon, South Korea; ^3^ Biomedical Mathematics Group, Institute for Basic Science, Daejeon, South Korea; ^4^ Research Institute of Pharmaceutical Sciences, College of Pharmacy, Kyungpook National University, Daegu, South Korea

**Keywords:** pharmacokinetic modeling, physiologically based pharmacokinetic (PBPK) model, lumping method, compartment model, compatibility

## Abstract

Pharmacokinetic (PK) modeling is a useful method for investigating drug absorption, distribution, metabolism, and excretion. The most commonly used mathematical models in PK modeling are the compartment model and physiologically based pharmacokinetic (PBPK) model. Although the theoretical characteristics of each model are well known, there have been few comparative studies of the compatibility of the models. Therefore, we evaluated the compatibility of PBPK and compartment models using the lumping method with 20 model compounds. The PBPK model was theoretically reduced to the lumped model using the principle of grouping tissues and organs that show similar kinetic behaviors. The area under the concentration–time curve (AUC) based on the simulated concentration and PK parameters (drug clearance [*CL*], central volume of distribution [*Vc*], peripheral volume of distribution [*Vp*]) in each model were compared, assuming administration to humans. The AUC and PK parameters in the PBPK model were similar to those in the lumped model within the 2-fold range for 17 of 20 model compounds (85%). In addition, the relationship of the calculated *Vd/fu* (volume of distribution [*Vd*], drug-unbound fraction [*fu*]) and the accuracy of AUC between the lumped model and compartment model confirmed their compatibility. Accordingly, the compatibility between PBPK and compartment models was confirmed by the lumping method. This method can be applied depending on the requirement of compatibility between the two models.

## 1 Introduction

Pharmacokinetic (PK) modeling is a research technique for quantifying and predicting the kinetics of drugs (Daryaee and Tongee, 2019). This technique has contributed to a reduction in failure rate and an increase in success rate in drug discovery and development (Gobburu and Marroum, 2001; Garralda et al., 2017). The main mathematical models used in PK modeling are the compartment model and physiologically based PK (PBPK) model (Lin et al., 2016). The compartment model explains the fate of a drug in the body through compartmentalization of the whole body on the premise of kinetic homogeneity. The number of compartments in the body is determined using the rate of drug distribution in a model body. In general, one- and two-compartment models are used. The compartment models are relatively simple, but they can efficiently predict the concentration of drugs in blood. However, the physicochemical properties of the drug (e.g., solubility, partition coefficient, protein binding) and the physiological properties of tissue and organs (e.g., volume, blood flow) cannot be reflected in the model (Khojasteh et al., 2011a; Jones and Rowland, 2013; Ahmed, 2015; Southwood et al., 2018). In contrast, the PBPK model associates the blood flow with each tissue and organ in the body by expressing the anatomical and physiological characteristics of the body as well as the physicochemical properties of drugs to predict the *in vivo* kinetics of the drug (Edginton et al., 2008; Jones et al., 2009). The PBPK model describes the drug distribution rate through each tissue and organ using models of perfusion rate limited tissue and permeability rate limited tissue. In the perfusion rate limited tissue model, the factors affecting the time for drugs to reach steady state are tissue volume (*V*_*T*_), tissue blood flow (*Q*_*T*_), and tissue to plasma partition coefficient (*K*_*PT*_). The permeability rate constant of the drug is a major component to determine the time for drugs to reach steady state in the permeability rate limited tissue model (Espie et al., 2009; Khalil and Läer, 2011; Utembe et al., 2020). The PBPK model allows prediction of the drug blood concentration and tissue distribution for various conditions by reflecting the physicochemical properties of the drug, the physiological properties of tissues and organs, and the PK properties related to the drug (e.g., metabolism, tissue distribution) (Shin et al., 2015; U.S. Food and Drug Administration, 2020). However, the PBPK model is mathematically and structurally more complex than the compartment model, and therefore requires a large amount of varied data to secure sufficient predictive power (Gerlowski and Jain, 1983; Anderson, 1995; Charnick et al., 1995).

The lumped model, a version of the multi-compartment PBPK model with fewer compartments and reduced complexity, was developed to overcome these limitations of the PBPK model. Several methods have been suggested to reduce the complexity of the formulas and structures by simplifying the PBPK model. A lumped model can be created by grouping tissues and organs with similar dynamic patterns to reflect the physiological characteristics of the body (Bernareggi and Rowland, 1991). Alternatively, a mathematical transformation method can be used that groups tissues of the same eigenvalue by matrixing each tissue concentration to ultimately calculate the eigenvalue (Okino and Mavrovouniotis, 1998). It is possible to minimize the errors and bias in the model by simplifying the PBPK model with a mathematical transformation method (Coxson and Bischoff, 1987; Li and Rabitz, 1991). However, this is a simplified method based on the mathematical theory that does not reflect the physiological characteristics of the body and dynamic factors of the drug (Kuo and Wei, 1969; Watson et al., 1996).

**FIGURE 1:**
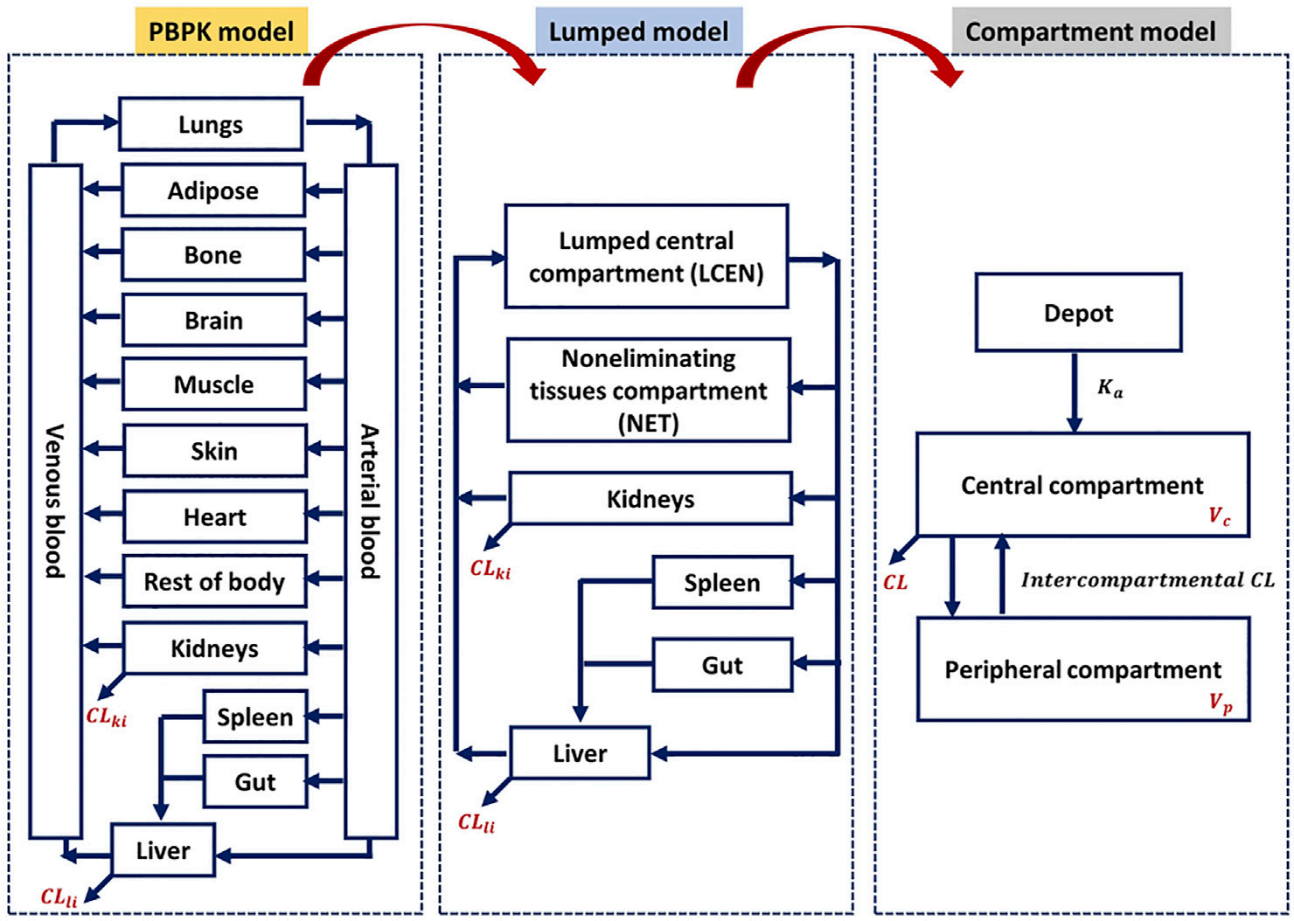
Schematic compatibility relationships among PBPK, lumped, and compartment models.

Although a few previous studies attempted to enhance model compatibility, including that between the PBPK and lumped models, in terms of mathematical concepts (Coxson and Bischoff, 1987; Li and Rabitz, 1991; Okino and Mavrovouniotis, 1998), an approach to evaluate the theoretical background across PBPK, lumped, and compartment models is still lacking, and no study thus far has shown its application to a real case. Therefore, we focused on evaluating the compatibility of the PBPK and compartment models using the lumping method and demonstrated with 20 real cases. The 20 model drugs were selected based on various ranges of systemic clearance, volume of distribution, therapeutic classification, and the biopharmaceutical drug disposition classification system (BDDCS) (Supplementary Table S1). Additionally, we selected some drugs using the PBPK model code developed for simulation. Although it is important to assess the concentrations of diverse compounds in the tissues and blood, there have been few studies of the PK characteristics of a wide range of compounds using different models. The objective of this study was to simplify of the model and verify of compatibility between the models for various drugs (Figure 1).

## 2 Materials and methods

### 2.1 Drugs and software

We selected 20 previously approved drugs for this study. PBPK models for these 20 model compounds were established as described in the literature (Supplementary Tables S2, S3). The R package mrgsolve (version 0.9.2, Metrum Research Group, Tariffville, CT. United States) was used to simulate model compounds, and non-compartment analysis (NCA) was performed using Phoenix WinNonlin (version 8.1; Certara, Princeton, NJ, United States) to calculate the area under the concentration-time curve (AUC).

### 2.2 PBPK modeling approach

**FIGURE 2:**
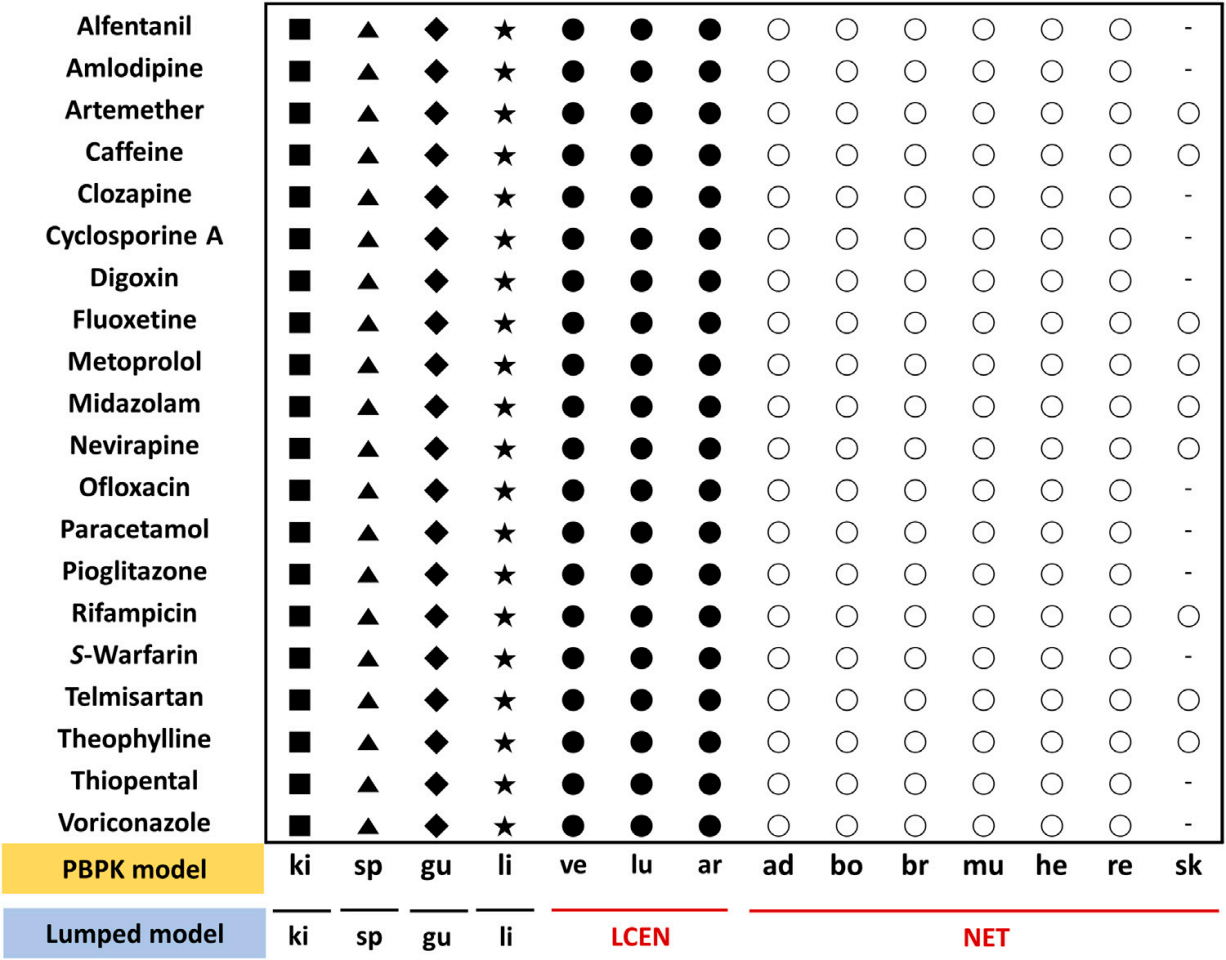
Assignment of tissues of the PBPK model to the lumped compartments of the lumped model for 20 model compounds. The lumped central compartment (LCEN) is represented by black circles and the non-eliminating tissues compartment (NET) is represented by white circles. The number of different symbols for a given compound corresponds to the number of compartments in the lumping model. Abbreviations ad, adipose; ar, arterial blood; bo, bone; br, brain; gu, gut; he, heart; ki, kidney; li, liver; lu, lung; mu, muscle; re, rest of body; sk, skin; sp, spleen; ve, venous blood.

This study applied the PBPK models to the model compounds by dividing various tissues and organs into compartments (Figure 2). In general, perfusion rate limited tissue models have been used for the tissue distribution in PBPK models. Therefore, this type of model was used (Jones et al., 2006; Peters and Hultin, 2008; Chen et al., 2012; Sinha et al., 2012). The physiological data (tissue volume, tissue blood flow) and input parameters (hepatic clearance, renal clearance, absorption rate constant, drug-unbound fraction, blood to plasma ratio) used in the model compound PBPK models are summarized in Supplementary Tables S2, S3, respectively. Every model we used was confirmed its validity by the sensitivity analysis and a goodness-of-fit (GOF), reported on relevant literatures. References are listed in Supplementary Table S3.

The following differential equations Eqs 1–3 were used to describe the changes in drug concentrations in arterial blood, venous blood, and lung, respectively:

$$V_{A}\times \frac{dC_{A\;}}{dt}=Q_{lu}\times \left(\frac{C_{lu}}{\frac{Kp_{lu}}{BP}}-C_{A}\right)$$ (1)

$$V_{V}\times \frac{dC_{V\;}}{dt}=\sum_{T}\left(Q_{T}\times \frac{C_{T}}{\frac{Kp_{T}}{BP}}\right)-Q_{lu}\times C_{V}$$ (2)

$$V_{lu}\times \frac{dC_{lu\;}}{dt}=Q_{lu}\times \left(C_{V}-\frac{C_{lu}}{\frac{Kp_{lu}}{BP}}\right)$$ (3)

where *V*_*A*_ is the arterial volume, *C*_*A*_ is the arterial drug concentration, *Q*_*lu*_ is the lung blood flow, *C*_*lu*_ is the lung drug concentration, *K*_*Plu*_ is the lung to plasma partition coefficient, *BP* is the blood to plasma ratio, *V*_*V*_ is the venous volume, *C*_*V*_ is the venous drug concentration, *Q*_*T*_ is the tissue blood flow, *C*_*T*_ is the tissue drug concentration, *K*_*PT*_ is the tissue to plasma partition coefficient, *V*_*lu*_ is the lung volume, and *C*_*lu*_ is the drug concentration in the lung. Note that in Eq. 2 for drug concentration in the venous blood, lung tissues that did not take up the drug through venous blood were excluded from calculating the uptake drug concentration (Elmokadem et al., 2019).

In tissues that do not eliminate drugs (e.g., adipose, bone, muscle), drug concentrations can be expressed by differential equations that show the differences between the uptake drug concentrations reflecting the tissue blood flow and output drug concentrations reflecting *Q*_*T*_ and *K*_*PT*_ as follows (Elmokadem et al., 2019):

$$V_{T}\times \frac{dC_{T\;}}{dt}=Q_{T}\times \left(C_{A}-\frac{C_{T}}{\frac{Kp_{T}}{BP}}\right)$$ (4)

where *V*_*T*_ is the tissue volume, *C*_*T*_ is the tissue drug concentration, *Q*_*T*_ is the tissue blood flow, *C*_*A*_ is the arterial drug concentration, *K*_*PT*_ is the tissue to plasma partition coefficient, and *BP* is the blood to plasma ratio.

The changes in drug concentrations in the tissues where drugs are eliminated, such as the liver and kidney, are described as follows (Yanni et al., 2010):

$$V_{T}\times \frac{dC_{T\;}}{dt}\;=Q_{T}\times \left(C_{A}-\frac{C_{T}}{\frac{Kp_{T}}{BP}}\right)-fu\times CL_{T}\times \frac{C_{T}}{\frac{Kp_{T}}{BP}}$$ (5)

where *V*_*T*_ is the tissue volume, *C*_*T*_ is the tissue drug concentration, *Q*_*T*_ is the tissue blood flow, *C*_*A*_ is the arterial drug concentration, *K*_*PT*_ is the tissue to plasma partition coefficient, *BP* is the blood to plasma ratio, *fu* is the drug-unbound fraction, and *CL*_*T*_ is the total tissue clearance. Hepatic clearance (*CL*_*hep*_) and renal clearance (*CL*_*ki*_) were obtained from the literature as described in Supplementary Table S3.

### 2.3 Lumped modeling approach

Lumped models were developed as described previously (Nestorov et al., 1998)*.* All tissues and organs compartmentalized in the PBPK model were grouped into six compartments based on similar physiological characteristics (Figure 2). Arterial blood, venous blood, and lungs were lumped into the lumped central compartment (LCEN). The tissues that did not eliminate drugs, such as adipose, bone, brain, muscle, heart, rest of the body, and skin, were lumped into the non-eliminating tissues compartment (NET). However, the tissues that eliminated drugs, such as liver and kidney, as well as the spleen and intestinal tract that are connected to the liver, were not lumped.

As described in Eqs 1–4, the main factors determining drug concentrations in blood and tissues in the PBPK model are *V*_*T*_, *Q*_*T*_, and *K*_*PT*_. These factors, therefore, were calculated using the following equations and applied in the lumped model. Volume (*V*_*Lump*_) and blood flow (*Q*_*Lump*_) in the lumped compartments were calculated as the sum of those of the lumped tissues. For the tissue-to-plasma partition coefficient (*K*_*Lump*_) in the lumped compartments, the sum of the partition coefficients reflecting the volume of tissues to be lumped was divided by the volume of the lumped compartment (Supplementary Tables S4, S5) (Nestorov et al., 1998; Pilari and Huisinga, 2010):

$$V_{Lump}\;=\underset{T}{\sum }V_{T}$$ (6)

$$Q_{Lump}\;=\underset{T}{\sum }Q_{T}$$ (7)

$$K_{Lump}\;=\frac{1}{V_{Lump}}\underset{T}{\sum }\;\left(V_{T}\times Kp_{T}\right)$$ (8)

where *V*_*Lump*_ is the volume in the lumped compartment, *V*_*T*_ is the tissue volume, *Q*_*Lump*_ is the blood flow in the lumped compartment, *Q*_*T*_ is the tissue blood flow, *K*_*Lump*_ is the tissue to plasma partition coefficient in the lumped compartment, and *K*_*PT*_ is the tissue to plasma partition coefficient.

The drug concentrations in LCEN and NET were calculated using Eqs 9, 10, respectively, as follows (Elmokadem et al., 2019):

$$V_{LCEN}\times \frac{dC_{LCEN\;}}{dt}\;=\underset{T}{\sum }\left(Q_{T}\times \frac{C_{T}}{\frac{Kp_{T}}{BP}}\right)-Q_{LCEN}\times C_{LCEN}$$ (9)

$$V_{NET}\times \frac{dC_{NET\;}}{dt}\;=Q_{NET}\times \left(C_{LCEN}-\frac{C_{NET}}{\frac{Kp_{NET}}{BP}}\right)$$ (10)

where *V*_*LCEN*_ is the volume in the LCEN, *C*_*LCEN*_ is the drug concentration in the LCEN, *Q*_*T*_ is the tissue blood flow, *C*_*T*_ is the tissue drug concentration, *K*_*PT*_ is the tissue to plasma partition coefficient, *BP* is the blood to plasma ratio, *Q*_*LCEN*_ is the blood flow in the LCEN, *V*_*NET*_ is the volume in the NET, *C*_*NET*_ is the drug concentration in the NET, *Q*_*NET*_ is the blood flow in the NET, and *K*_*PNET*_ is the tissue to plasma partition coefficient in the NET. Note that in Eq. 9 for calculation of the drug concentration in the LCEN, the systematic circulation tissues that did not receive blood supply from the venous blood were excluded from the sum for normalization using the *V*_*T*_, *Q*_*T*_, and *K*_*PT*_ of each tissue.

### 2.4 Compartment model approach

The one- or two-compartment model was used for model compounds, whereas drug clearance (*CL*), central volume of distribution (*Vc*), peripheral volume of distribution (*Vp*), inter-compartmental clearance (*Q*), and absorption rate constant (*Ka*) values were obtained from the literature, as described in Supplementary Table S6. We performed model validation, according to the relevant literature, by checking the GOF plot and conducting a visual predictive check (VPC). References are listed in Supplementary Table S6.

### 2.5 Theoretical considerations of compatibility among PBPK, lumped, and compartment models

Considering the origin of each PK model, PK models, what it mentioned above, could be expected by compatibility based on meaning of mathematical and biological assumptions.

For instance, the total clearance in the PK model could be represented by Eq. 11 (Cao and Jusko, 2012).

$$CL_{T}=\;CL_{hep\;}+\;CL_{ki\;}+\;CL_{others\;}$$ (11)

where *CL*_*T*_ is the total tissue clearance, *CL*_*hep*_ is the hepatic clearance, *CL*_*ki*_ is the renal clearance, *CL*_*others*_ is the sum of tissue clearances except liver and kidney.

Despite the difference of the way to *CL*_*T*_ among PBPK, lumped, and compartment model, *CL*_*T*_ should be approximated by theoretically true clearance regardless of way of estimation. In general, *CL*_*T*_ could be estimated based on blood concentrations, so we could suppose that *CL*_*T*_, that is calculated by sum of *CL*_*hep*_ *, CL*_*ki*_ and *CL*_*others*_ obtained by PBPK or lumped model, should be similar with estimation of *CL*_*T*_ value from compartment model.

In addition, above mentioned approaches could be acceptable in case of drug distribution related with volume of distribution to tissue. The rate of drug distribution to liver may be defined in the PBPK and lumped models as Eq. 12 (Cao and Jusko, 2012).

$$V_{li}\times \frac{dC_{li\;}}{dt}\;=Q_{li}\times \left(C_{A}-\frac{C_{li}}{\frac{Kp_{li}}{BP}}\right)-fu\times CL_{hep}\times \frac{C_{li}}{\frac{Kp_{li}}{BP}}$$ (12)

where *V*_*li*_ is the liver volume, *C*_*li*_ is the liver drug concentration, *Q*_*li*_ is the liver blood flow, *C*_*A*_ is the arterial drug concentration, *K*_*Pli*_ is the liver to plasma partition coefficient, *BP* is the blood to plasma ratio, *fu* is the drug-unbound fraction, and *CL*_*hep*_ is the hepatic clearance.

Since the lumped model was focused on merging the compartment where it has similar biological characteristics in comparison with PBPK, the compatibility between them could be easily explained.

Moreover, the theoretical compatibility among PBPK, lumped, and compartment model could be explained by additional assumption. For example, the well-stirred assumption of the hepatic compartment may be also applicable. The PBPK and lumped models with hepatic compartment can be related to clearance concepts of compartment model, assuming the well-stirred model as follows (Cao and Jusko, 2012):

$$CL_{hep}=\;Q_{li}\;\times \frac{fu\times CL_{int}}{fu\times CL_{int}+Q_{li}}\;\;$$ (13)

where *CL*_*hep*_ is the hepatic clearance, *Q*_*li*_ is the liver blood flow, *fu* is the drug-unbound fraction, and *CL*_*int*_ is the intrinsic clearance.

Therefore, *CL*_*T*_ and apparent *Vd* of drugs, having mainly distributed into liver and eliminated by liver, could be approximately calculated with *fu* and *CL*_*hep*_.

### 2.6 Evaluation of compatibility among PBPK, lumped, and compartment models

Simulations were performed 1,000 times to compare the compatibility of the models. The therapeutic dose of each drug was administered orally in a single dose to adults having similar weight, who were then followed up at various simulation intervals (e.g., 0–48 h or 0–312 h) depending on the drugs and their dosing amounts to determine the drug concentrations under the same experimental conditions in the three models. To compare the drug concentrations in the blood and tissues between each model, the AUC using NCA was utilized as the PK parameter for exposure (Scheff et al., 2011). However, the maximum blood concentration (C_max_), which is related to absorption, was excluded because this study was performed to examine whether drug distribution, metabolism, and excretion could be lumped. Moreover, it is well known that the variation of C_max_ is 50%–60% higher than that of the AUC (Endrenyi and Yan, 1993). Therefore, key parameters of PK, such as drug *CL*, *Vc*, and *Vp*, were compared (Benet, 1984). To compare the AUC and PK parameters, we used the 2-fold range criteria typically used as the acceptance criteria for the PBPK model (Sager et al., 2015). Additionally, we have performed statistical analysis to compare the AUC and clearance among PBPK, lumped, and compartment model. Those results are described in Tables 1–3. Comparison of AUC and PK parameters was performed according to the following steps:

**TABLE 1:** Comparison of AUC parameters of central compartment in PBPK, lumped, and compartment models for 20 compounds.

Model	PBPK model	Lumped model	Compartment model
Tissue, compartment	Lungs, arterial blood, venous blood	Lumped central compartment (LCEN)	Central compartment
Parameter (unit)	Average of tissue AUC_last_ (mg•h/L)	AUC_last_ at LCEN (mg•h/L)	AUC_last_ at central compartment (mg•h/L) (2-fold range)
Compound	(2-fold range)	(2-fold range)	
Alfentanil	0.348 (0.174-0.697)	0.351 (0.175-0.702)	0.585 (0.293-1.171)
Amlodipine	7.598 (3.799-15.195)	0.505 (0.253-1.011)^*^	0.496 (0.248-0.993)^#^
Artemether	2.608 (1.304-5.216)	2.877 (1.438-5.754)	0.173 (0.087-0.346)^+,#^
Caffeine	1.349 (0.674-2.698)	1.482 (0.741-2.963)	0.953 (0.477-1.906)
Clozapine	130.033 (65.017-260.066)	12.823 (6.411-25.645)^*^	14.172 (7.086-28.344)^#^
Cyclosporine A	21.776 (10.888-43.552)	12.285 (6.143-24.571)	18.363 (9.181-36.726)
Digoxin	0.056 (0.028-0.111)	0.060 (0.030-0.120)	0.030 (0.015-0.060)
Fluoxetine	2.736 (1.368-5.471)	1.926 (0.963-3.851)	4.511 (2.256-9.022)
Metoprolol	0.987 (0.494-1.974)	1.074 (0.537-2.147)	0.630 (0.315-1.260)
Midazolam	1.005 (0.503-2.010)	0.977 (0.489-1.954)	0.098 (0.049-0.196)
Nevirapine	146.303 (73.151-292.605)	164.225 (82.113-328.450)	203.575 (101.787-407.150)
Ofloxacin	50.220 (25.110-100.440)	53.246 (26.623-106.492)	55.173 (27.586-110.345)
Paracetamol	28.650 (14.325-57.300)	33.827 (16.914-67.655)	67.964 (33.982-135.929)
Pioglitazone	2.368 (1.184-4.735)	3.201 (1.600-6.402)	5.529 (2.765-11.058)
Rifampicin	153.468 (76.734-306.937)	79.553 (39.777-159.106)	65.084 (32.542-130.168)
*S*-Warfarin	28.075 (14.037-56.150)	35.292 (17.646-70.584)	30.006 (15.003-60.012)
Telmisartan	760.097 (380.049-1,520.195)	986.822 (493.411-1973.643)	0.707 (0.353-1.414)^+,#^
Theophylline	468.329 (234.164-936.658)	554.051 (277.026-1,108.102)	65.239 (32.620-130.479)
Thiopental	1,688.083 (844.042-3,376.166)	1819.869 (909.935-3,639.738)	3,015.313 (1,507.657-6,030.626)
Voriconazole	43.587 (21.794-87.174)	46.360 (23.180-92.720)	63.400 (31.700-126.800)

AUC, area under the concentration-time curve. ^*,+, #^ marks are attached after compound name if the AUC, parameters among PBPK, model, lumped model, and compartment model are significantly different (*p* < 0.05) (post-hoc analysis: ^*^PBPK, model and lumped model, ^#^PBPK, model and compartment model, ^+^lumped model and compartment model).

Step 1 Each model was built based on the parameters described in the literature (e.g., *CL*, *Vc*, *Vp*).

Step 2 Comparison of AUC obtained from drug concentrations in tissues and blood simulated using each model.

Step 3 Comparison of *CL* and *Vc* between models.

Step 4 Comparison of *Vp* between models (*Vp* in PBPK and lumped models were calculated using Eqs 14, 15, respectively).

$$V_{p\;in\;PBPK\;model\;}=\bar{\frac{V_{T}\;\times \;Kp_{T}}{Body\;weight}}$$ (14)

$$V_{p\;in\;lumped\;model\;}=\frac{\bar{V_{T}\;}\times \bar{\;Kp_{T}}}{Body\;weight}$$ (15)

where *V*_*p*_ is the peripheral volume of distribution, *V*_*T*_ is the tissue volume, and *K*_*PT*_ is the tissue to plasma partition coefficient.

To further approach the compatibility among the three models, we estimated the empirical relationship of the calculated *Vd/fu* and the accuracy of the AUC between the lumped and compartment models.

Step 5 Estimation of empirical relations between lumped and compartment models for the ratio between the volume of distribution (*Vd*, where *Vd* is typically assumed to be the sum of *Vc* and *Vp*) and the drug-unbound fraction (*fu*). Note that the descriptors *Vc*, *Vp*, and *fu* are shown in Supplementary Tables S3, S7, S8, respectively.

Step 6 Estimation of the accuracy of the AUC as follows:

$$Theoritical\;AUC_{last\;at\;central\;compartment}=\;AUC_{last\;at\;LCEN\;\;}\times \;\frac{CL_{in\;lumped\;model}}{CL_{in\;compartment\;model}}\;\;$$ (16)

$$The\;accuracy\;of\;AUC=\;\frac{AUC_{last\;at\;central\;compartment\;\;}}{Theoritical\;AUC_{last\;at\;central\;compartment\;\;}}\;\;$$ (17)

## 3 Results

### 3.1 Comparison of AUCs

To confirm the model development steps, the performances of all models were confirmed by comparison between simulated and reported PK profiles. The distribution rate in PBPK model is assumed to be governed by rapid equilibrium. Based on this assumption, the average value of each tissue AUC in the PBPK model was compared with each compartment in lumped and the compartment model.

The AUCs in the central compartment of each model are shown in Table 1. Those for arterial blood, venous blood, and lungs in the PBPK model were similar to those in the LCEN of the lumped model within a range of 2-fold for 18 of the 20 model compounds (90%). For clozapine and amlodipine, however, the values for differed considerably between the PBPK model and the LCEN of the lumped model. The AUCs of clozapine and amlodipine of the PBPK model were 130.033 mg h/L and 7.598 mg h/L, respectively. These results differed from the estimates of 12.823 mg h/L and 0.505 mg h/L, respectively, in the lumped model. Furthermore, the AUCs in LCEN of the lumped model were similar to those of the compartment model within the range of 2-fold for 14 of the 20 model compounds (70%); the exceptions were midazolam, telmisartan, paracetamol, artemether, fluoxetine, and theophylline.

The AUCs in the peripheral compartment are shown in Table 2. In adipose, bone, brain, muscle, heart, rest of the body, and skin, where the drugs were not eliminated, the AUCs after lumping were included in the AUCs before lumping for 19 of the 20 model compounds (95%). In the case of metoprolol, however, the AUC of the NET deviated from the 2-fold range between the PBPK and lumped models with values of 5.476 mg h/L and 2.644 mg h/L, respectively. Furthermore, the AUCs in the NET of the lumped model differed from those of the compartment model for most two-compartment model compounds (6 of 9, 66.7%).

**TABLE 2:** Comparison of AUC parameters of peripheral compartment in PBPK, lumped, and compartment models for 20 compounds.

Model	PBPK model	Lumped model	Compartment model
Tissue, compartment	Adipose, bone, brain, muscle, skin, heart, rest of body	Non-eliminating tissues compartment (NET)	Peripheral compartment
Parameter (unit)	Average of tissue AUC_last_ (mg•h/L) (2-fold range)	AUC_last_ at NET (mg•h/L) (2-fold range)	AUC_last_ at peripheral compartment (mg•h/L) (2-fold range)
Compound			
Alfentanil	1.772 (0.886-3.544)	0.994 (0.497-1.987)	-
Amlodipine	9.979 (4.989-19.957)	11.920 (5.960-23.840)	-
Artemether	27.217 (13.609-54.435)	19.090 (9.545-38.180)	-
Caffeine	0.901 (0.450-1.801)	0.638 (0.319-1.276)	-
Clozapine	142.308 (71.154-284.617)	134.485 (67.242-268.969)	-
Cyclosporine A	17.777 (8.888-35.553)	12.601 (6.300-25.202)	-
Digoxin	0.077 (0.039-0.154)	0.057 (0.028-0.113)	0.030 (0.015-0.059)
Fluoxetine	20.178 (10.089-40.356)	13.400 (6.700-26.799)	-
Metoprolol	5.476 (2.738-10.952)	2.644 (1.322-5.288)	-
Midazolam	8.860 (4.430-17.720)	5.189 (2.594-10.377)	0.100 (0.050-0.201)^+,#^
Nevirapine	315.957 (157.979-631.914)	208.837 (104.418-417,674)	202.734 (101.367-405.467)
Ofloxacin	34.537 (17.269-69.074)	29.840 (14.920-59.680)	55.224 (27.612-110.449)
Paracetamol	18.966 (9.483-37.931)	19.120 (9.560-38.240)	68.139 (34.070-136.278)^+,#^
Pioglitazone	0.348 (0.174-0.696)	0.312 (0.156-0.624)	3.002 (1.501-6.005)^+,#^
Rifampicin	139.803 (69.901-279.605)	163.653 (81.827-327.307)	-
*S*-Warfarin	6.697 (3.348-13.394)	5.905 (2.953-11.810)	-
Telmisartan	164.733 (82.367-329.466)	123.565 (61.783-247.130)	0.694 (0.347-1.388)^+,#^
Theophylline	291.616 (145.808-583.232)	279.114 (139.557-558.228)	-
Thiopental	11,940.270 (5,970.135-23880.540)	7,818.454 (3,909.227-15636.908)	2,773.715 (1,386.858-5,547.430)
Voriconazole	260.225 (130.113-520.450)	273.008 (136.504-546.016)	63.054 (31.527-126.108)

AUC, area under the concentration-time curve. ^*,+, #^ marks are attached after compound name if the AUC, parameters among PBPK, model, lumped model, and compartment model are significantly different (*p* < 0.05) (post-hoc analysis: ^*^PBPK, model and lumped model, ^#^PBPK, model and compartment model, ^+^lumped model and compartment model).

### 3.2 Comparison of PK parameters

Next, we compared the *CL*, *Vc*, and *Vp*.

*CL* is the sum of clearance in the liver and kidneys, which was not significantly different within the 2-fold range between the three models for 19 of the 20 model (95%), the exception being alfentanil for which *CL* deviated from the 2-fold range between the three models with values of 0.555 L/h/kg and 0.209 L/h/kg in the PBPK/lumped models and the compartment model, respectively (Table 3).

**TABLE 3:** Comparison of *CL* parameters in PBPK, lumped, and compartment models for 20 compounds.

Parameter (unit)	*CL* (L/h/kg)		
ModelCompound	PBPK model (2-fold range)	Lumped model (2-fold range)	Compartment model (2-fold range)
Alfentanil	0.555 (0.278-1.111)	0.555 (0.278-1.111)	0.209 (0.104-0.417)
Amlodipine	0.408 (0.204-0.816)	0.408 (0.204-0.816)	0.255 (0.127-0.509)
Artemether	13.333 (6.667-26.667)	13.333 (6.667-26.667)	16.436 (8.218-32.873)
Caffeine	0.134 (0.067-0.268)	0.134 (0.067-0.268)	0.094 (0.047-0.189)
Clozapine	0.401 (0.201-0.803)	0.401 (0.201-0.803)	0.313 (0.156-0.626)
Cyclosporine A	0.420 (0.210-0.841)	0.420 (0.210-0.841)	0.459 (0.229-0.918)
Digoxin	0.136 (0.068-0.273)	0.136 (0.068-0.273)	0.222 (0.111-0.444)
Fluoxetine	0.351 (0.175-0.702)	0.351 (0.175-0.702)	0.208 (0.104-0.416)
Metoprolol	3.250 (1.625-6.500)	3.250 (1.625-6.500)	2.821 (1.411-5.643)
Midazolam	0.540 (0.270-1.080)	0.540 (0.270-1.080)	0.896 (0.448-1.791)
Nevirapine	0.022 (0.011-0.044)	0.022 (0.011-0.044)	0.015 (0.008-0.031)
Ofloxacin	0.160 (0.080-0.320)	0.160 (0.080-0.320)	0.132 (0.066-0.265)
Paracetamol	0.270 (0.135-0.540)	0.270 (0.135-0.540)	0.215 (0.108-0.430)
Pioglitazone	0.068 (0.034-0.137)	0.068 (0.034-0.137)	0.035 (0.018-0.071)
Rifampicin	0.142 (0.071-0.283)	0.142 (0.071-0.283)	0.163 (0.081-0.326)
*S*-Warfarin	0.003 (0.001-0.006)	0.003 (0.001-0.006)	0.002 (0.001-0.004)
Telmisartan	0.800 (0.400-1.600)	0.800 (0.400-1.600)	0.980 (0.490-1.960)
Theophylline	0.045 (0.023-0.091)	0.045 (0.023-0.091)	0.054 (0.027-0.108)
Thiopental	0.189 (0.095-0.378)	0.189 (0.095-0.378)	0.114 (0.057-0.229)
Voriconazole	0.106 (0.053-0.212)	0.106 (0.053-0.212)	0.088 (0.044-0.176)

*CL*, clearance. ^*,+, #^ marks are attached after compound name if the AUC, parameters among PBPK, model, lumped model, and compartment model are significantly different (*p* < 0.05) (post-hoc analysis: ^*^PBPK, model and lumped model, ^#^PBPK, model and compartment model, ^+^lumped model and compartment model).

*Vc* where the drug is rapidly and homogeneously distributed was equal to the total tissue volume and was similar between the PBPK and lumped models. However, *Vc* of the lumped model differed from that in the central compartment of the compartment model for most two-compartment model compounds (12 of 20, 60.0%) (Supplementary Table S7).

*Vp* where the drugs are distributed in a slow and heterogeneous manner was similar within the 2-fold range between the PBPK and lumped models for 19 of 20 model compounds (95%), the exception being metoprolol for which *Vp* deviated from the 2-fold range between the PBPK and lumped models with values of 0.278 L/kg and 0.593 L/kg, respectively (Supplementary Table S8). In addition, the volume of distribution in the peripheral compartment of the lumped model differed from that in the peripheral compartment of the compartment model for most two-compartment model compounds (8 of 9, 88.9%).

**FIGURE 3:**
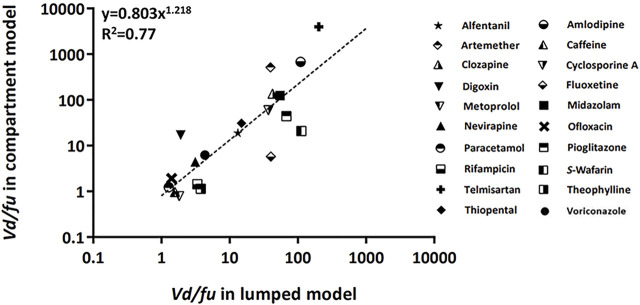
Relationship of the calculated *Vd*/*fu* between lumped and compartment models. The dashed line was fitted to the indicated relationship (*y* = *ax* ^*b*^). Abbreviations *fu*, drug-unbound fraction; *Vd*, volume of distribution.

The empirical relations of the calculated *Vd/fu* between the lumped and compartment models are shown in Figure 3 (Khojasteh et al., 2011b). The concept of this post hoc analysis which compares with the *Vd/Fu* value between two models was based on the fact that the apparent ideal volume of distribution was close to that of the unbound drug fraction. Furthermore, the protein binding effect on the volume of distribution could be more significant in the peripheral compartment than in the central compartment (Holford and Yim, 2016). Therefore, we had to pay attention that the volume of the peripheral can be distributed to the tissue in inverse proportion to the value of the unbound drug fraction. Using this analysis, the calculated *Vd*/*fu* in the lumped model was correlated with the calculated *Vd*/*fu* in the compartment model.

**FIGURE 4:**
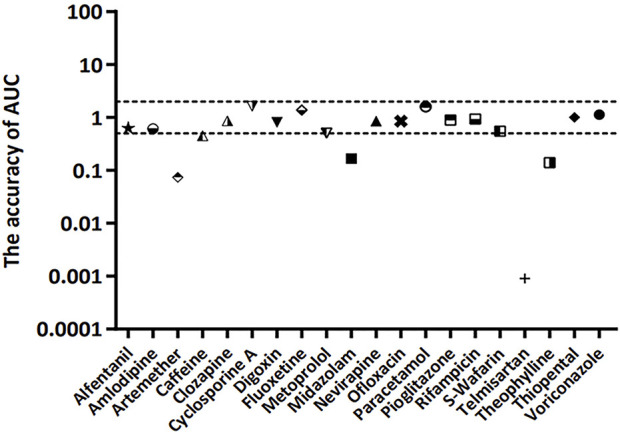
Accuracy of AUC for 20 model compounds. The accuracy of AUC was calculated using Eqs 16, 17. The dashed line represents 2-fold deviation.

The accuracy of the AUC between the lumped and compartment models was assessed using Eqs 16, 17. The approach was based on the fact that the *CL* ratio between two models is used for model-to-model conversion. The compartmental AUC was theoretically calculated using Eq. 16, and the accuracy of the AUC was estimated using Eq. 17. The compatibility between the two models was confirmed when the accuracy of the AUC was approximately one. The accuracy of AUC was found to be within the 2-fold range for 15 of 20 model drugs (75%) (Figure 4).

## 4 Discussion

Despite the availability of user-friendly software to resolve the technical issues, the barriers to entry for building and understanding full PBPK models have consistently posed difficulties for beginners in pharmacometrics. Therefore, a number of methods have been suggested to simplify full PBPK models, such as simplified PBPK and lumped PBPK modeling approaches. However, there is no precedent for implying or connecting these various PBPK models to traditional compartment models that have been used for many years. Here, we attempted to confirm the relations among the PBPK, lumped, and compartment models. Therefore, in this study, we demonstrated that it is possible to lump tissues and organs with similar physiological characteristics into the PBPK model for 20 model compounds and that the lumped and compartment models are compatible with the PBPK model.

For comparison of compatibility between the models, the AUC was compared between models as the PK parameter for exposure. The AUC in central and peripheral compartments of the PBPK model was similar to that of the lumped model within the 2-fold range for 17 of 20 model compounds (85%), with the exceptions being metoprolol, clozapine, and amlodipine. This indicated that the two models were compatible with regard to drug concentration. As shown in Tables 1, 2, however, the AUCs of metoprolol, clozapine, and amlodipine deviated from the 2-fold range between the PBPK and lumped models. Such AUC differences for metoprolol and clozapine have been reported previously due to their high PK variability (Ågesen et al., 2019; Lee et al., 2021). The AUC of central and peripheral compartment differed between lumped and compartment models for 9 of 20 model compounds (45%). This difference may have been attributable to the differences in how these methods reflect the volume of distribution. In the lumped model, the volume of each tissue is an important factor for predicting the drug concentration in tissues and blood. Overall, this volume was accurately reproduced by the model. In contrast, the volume of distribution is calculated based on the blood concentration in the compartment model. In particular, the distribution volume of the peripheral compartment may exhibit greater differences in the AUC due to the difficulties in reflecting the blood concentration. Moreover, the errors due to the lack of inclusion of the tissue to plasma distribution coefficients may also be responsible for these differences. Note that for telmisartan, the difference in the AUC may have been attributable to the non-linear PK characteristics and high individual differences in response to the drug (Stangier et al., 2000; U.S. Food and Drug Administration, 2009). In addition, the difference of C_max_ among the models were compared as well, however, the C_max_ were observed by the difference over 4-fold among the models in comparison with AUC because of reasons (e.g, large variation) as we stated in Section 2.6 (Supplementary Tables S9, S10).

To confirm the compatibility of the models, *CL*, *Vc*, and *Vp*, as the key parameters of PK, were compared between models. *CL* was similar within the 2-fold range between the three models for 19 of 20 model compounds (95%), with the exception being alfentanil. Thus, the three models were compatible for comparison of *CL*. However, the *CL* of alfentanil deviated from the 2-fold range between the three models (0.555 L/h/kg for PBPK model and lumped models and 0.209 L/h/kg for the compartment model). This difference may have been attributable to the high inter-individual differences in clearance of alfentanil; alfentanil is eliminated mainly by hepatic metabolism, and the clearance varied by more than 4-fold, resulting in large inter-individual differences probably due to variation in the hepatic metabolic capacity. Furthermore, inter-individual differences in protein binding may also influence clearance (Henthorn et al., 1985; Persson et al., 1988). The volumes of distribution in the central and peripheral compartments were similar between PBPK and lumped models for 19 of 20 model compounds (95%), with the exception being metoprolol for which the volume of distribution in the peripheral compartment deviated from the 2-fold range between the two models (0.278 L/kg for the PBPK model and 0.593 L/kg for the lumped model). As mentioned above, this difference may have been attributable to the high variability of metoprolol PKs (Ågesen et al., 2019). The volumes of distribution in the central and peripheral compartment were different between lumped and compartment models. In interpreting this finding, it is necessary to consider that the deviation in the volume of distribution may be greater than clearance. Furthermore, the compatibility of volume of distribution was not confirmed due to the differences in the tissue to plasma partition coefficients and methods of reflecting blood flow velocity of each tissue organ.

To further assess the compatibility among the three models, we used the empirical relationship of the calculated *Vd/fu* and the accuracy of AUC between lumped and compartment models. According to these approaches, the compatibility of PBPK, lumped, and compartment models for 20 model compounds was examined. Although telmisartan is outside the criteria range due to its non-linear PK characteristics and individual differences, the overall results indicated that the three models were compatible in terms of PK parameters (Stangier et al., 2000; U.S. Food and Drug Administration, 2009). Furthermore, the drug concentration of each tissue in the PBPK model could be indirectly estimated by using the drug concentration of the lumped and compartment models when the three models were compatible for drug concentration (e.g., voriconazole). In the PBPK model, the drug movement was determined by *V*_*T*_, *Q*_*T*_, and *K*_*PT*_. These parameters were calculated using the lumping equation and further applied to the lumped and compartment models. The drug concentration in each tissue of the PBPK model can be calculated using Eq. 18 (Pilari and Huisinga, 2010).

$$C_{T}\;=C_{p}\times \frac{V_{p\;in\;compartment\;model\;}}{V_{p\;in\;lumped\;model\;}}\times \frac{Kp_{T}}{K_{Lump}}$$ (18)

The drug concentration of each tissue in the PBPK model could be predicted by applying Eq. 18. Overall, there were no differences in the AUC of the PBPK model and AUC calculated using Eq. 18 (Supplementary Table S11).

The following limitations must be taken into consideration in interpretation of the findings of this study. Only lumping of the perfusion rate limited tissue model was assessed in the PBPK model based on a single dose of model compounds and a single administration route. In future studies, other administration routes and doses of model compounds, as well as other drugs for permeability rate-limited tissue models, would elucidate new pathways for the lumped model and would help to establish better compatibility between the three models. Moreover, simplification of the model and verification of compatibility between the models for other drugs would facilitate the prediction of drug profiles in tissues using a relatively simple model.

In summary, we evaluated the compatibility between PBPK and compartmental PK models using the lumping method. This study suggested that this lumping method may be useful to provide a simplified PBPK model. Construction of a lumped model may also be possible that can be assessed relative to the compartment model.

## 5 Conclusion

This study evaluated the compatibility between the PBPK and compartment models using the lumping method with 20 model compounds, and further approaches were attempted to determine a theoretical method to establish compatibility between the models. The lumping method is considered to assess the models’ compatibility, suggesting the reliability of the PK parameters of the PBPK and compartment models. The lumping method may be further utilized to develop and extend the PBPK and compartment models. Additionally, the lumping method approach with the PBPK model uses a relatively small amount of data and facilitates access to the compartment model. Hence, this approach could help for pharmacometricians gain a deeper understanding of the associations and alignments between the models.

## Data Availability

The original contributions presented in the study are included in the article/Supplementary Material, further inquiries can be directed to the corresponding authors.
