# Supplementary material for: A compatibility evaluation between the physiologically based pharmacokinetic (PBPK) model and the compartmental PK model using the lumping method with real cases
Source: Front Pharmacol. 2022 Aug 12;13:964049. doi: 10.3389/fphar.2022.964049 (PMC9413202; doi:10.3389/fphar.2022.964049)
Supplement: Supplementary file 1 [file DataSheet1.docx]

Supplementary Material

# Supplementary Figures and Tables

Table S1. Compound list (Benet et al., 2011; Druginfo, 2000; Medindia, 1999; Varma et al., 2012)

| Compound | Therapeutic classification | BDDCS |
| --- | --- | --- |
| Alfentanil | Narcotic analgesics | Class 1 |
| Amlodipine | Calcium channel blockers | Class 1 |
| Artemether | Anti-malarials | Class 2 |
| Caffeine | Central nervous system stimulants | Class 1 |
| Clozapine | Antipsychotics | Class 2 |
| Cyclosporine A | Immunosuppressants | Class 2 |
| Digoxin | Drugs for heart failure | Class 3 |
| Fluoxetine | Selective serotonin reuptake inhibitors | Class 1 |
| Metoprolol | Beta blockers | Class 1 |
| Midazolam | Hypnotics and sedatives, minor tranquilizer | Class 1 |
| Nevirapine | Antifungal agents | Class 2 |
| Ofloxacin | Quinolones | Class 3 |
| Paracetamol | Other analgesics and antipyretics | Class 1 |
| Pioglitazone | Thiazolidinediones | Class 2 |
| Rifampicin | Antitubercular agents | Class 2 |
| *S*-Warfarin | Anticoagulants | Class 2 |
| Telmisartan | Angiotensin II receptor antagonists | Class 2 |
| Theophylline | Respiratory relaxants | Class 1 |
| Thiopental | General anesthetics | Class 1 |
| Voriconazole | Antifungal agents | Class 2 |

BDDCS, biopharmaceutical drug disposition classification system.

**Reference**

Benet, L.Z., Broccatelli, F., and Oprea, T.I. (2011). BDDCS applied to over 900 drugs. *AAPS J*. 13, 519-547. doi: 10.1208/s12248-011-9290-9

Druginfo. (2000). https://www.druginfo.co.kr/ [Accessed January 10, 2021].

Medindia. (1999). https:// www.medindia.net/doctors/drug_information/ [Accessed January 10, 2021].

Varma, M.V., Gardner, I., Steyn, S.J., Nkansah, P., Rotter, C.J., Whitney-Pickett, C., et al. (2012). pH-Dependent solubility and permeability criteria for provisional biopharmaceutics classification (BCS and BDDCS) in early drug discovery. *Mol. Pharm*. 9, 1199-1212. doi: 10.1021/mp2004912

Table S2. Physiological input parameters for 20 compounds in the PBPK model (International Commission on Radiological Protection, 2002)

| Compound | Body weight (kg) | Tissue | *V_T_* (L) | *Q_T_* (L/h) |
| --- | --- | --- | --- | --- |
| Amlodipine, digoxin, ofloxacin, paracetamol, pioglitazone, *S*-warfarin, thiopental, voriconazole | 73 | Adipose | 18.200 | 19.500 |
|  |  | Bone | 10.500 | 19.500 |
|  |  | Brain | 1.450 | 46.800 |
|  |  | Muscle | 29.000 | 66.300 |
|  |  | Heart | 0.330 | 15.600 |
|  |  | Rest of body | 4.160^*^, 3.050 | 52.650^*^, 48.750 |
|  |  | Kidneys | 0.310 | 74.100 |
|  |  | Spleen | 0.150 | 11.700 |
|  |  | Gut | 1^*^, 2.11 | 58.800^*^, 62.400 |
|  |  | Liver | 1.800 | 25.350 (arterial), 95.550 (total)^*^  25.350 (arterial), 99.450 (total) |
|  |  | Lungs | 0.500 | 390.000 |
|  |  | Artery | 1.652 | 390.000 |
|  |  | Vein | 3.948 | 390.000 |
| Artemether, caffeine, fluoxetine, metoprolol, midazolam, nevirapine, rifampicin, telmisartan, theophylline | 60 | Adipose | 22.500 | 30.090 |
|  |  | Bone | 7.800 | 17.700 |
|  |  | Brain | 1.300 | 42.480 |
|  |  | Muscle | 17.500 | 42.480 |
|  |  | Skin | 2.300 | 17.700 |
|  |  | Heart | 0.250 | 17.700 |
|  |  | Rest of body | 1.075 | 30.090 |
|  |  | Kidneys | 0.275 | 60.180 |
|  |  | Spleen | 0.130 | 10.620 |
|  |  | Gut | 1.030 | 60.180 |
|  |  | Liver | 1.400 | 21.240 (arterial), 95.580 (total) |
|  |  | Lungs | 0.420 | 354.000 |
|  |  | Artery | 1.300 | 354.000 |
|  |  | Vein | 2.600 | 354.000 |
| Alfentanil, clozapine, cyclosporine A | 70 | Adipose | 18.200 | 19.500 |
|  |  | Bone | 10.500 | 19.500 |
|  |  | Brain | 1.450 | 46.800 |
|  |  | Muscle | 29.000 | 66.300 |
|  |  | Heart | 0.330 | 15.600 |
|  |  | Rest of body | 0.050 | 48.750 |
|  |  | Kidneys | 0.310 | 74.100 |
|  |  | Spleen | 0.150 | 11.700 |
|  |  | Gut | 2.11 | 62.400 |
|  |  | Liver | 1.800 | 25.350 (arterial), 99.450 (total) |
|  |  | Lungs | 0.500 | 390.000 |
|  |  | Artery | 1.652 | 390.000 |
|  |  | Vein | 3.948 | 390.000 |

*V_T_*, tissue volume; *Q_T_*, tissue blood flow. ^*^Physiological input parameters for voriconazole PBPK model.

**Reference**

International Commission on Radiological Protection. (2002). Basic anatomical and physiological data for use in radiological protection: Reference values. https://journals.sagepub.com/doi/pdf/10.1177/ANIB_32_3-4 [Accessed January 10, 2021].

Table S3. Input parameters for 20 compounds in the PBPK model

| Parameter (unit)  Compound | *CL_he_****_p_*** (L/h) | *CL_ki_* (L/h) | *K_a_* (1/h) | *f_u_* | *BP* | Source |
| --- | --- | --- | --- | --- | --- | --- |
| Alfentanil | 38.880 | 0.000 | 0.849 | 0.110 | 0.630 | Bower and Hull (1982)  metrumresearchgroup/PBPK_PC (2020) |
| Amlodipine | 28.000 | 1.800 | 0.800 | 0.025 | 0.596 | DrugBank Online (n.d.a)  Mukherjee et al. (2018) |
| Artemether | 800.000 | 0.000 | 0.500 | 0.046 | 0.800 | metrumresearchgroup/PBPK_PC (2020) |
| Caffeine | 8.000 | 0.038 | 2.180 | 0.681 | 0.980 | metrumresearchgroup/PBPK_PC (2020) |
| Clozapine | 28.100 | 0.000 | 2.220 | 0.055 | 0.845 | Schaber et al. (1998)  Ghoneim and Mansour (2020)  Lee et al. (2021) |
| Cyclosporine A | 29.400 | 0.029 | 1.659 | 0.036 | 1.620 | DrugBank Online (n.d.b)  Lee et al. (2007)  Yoon et al. (2019)  Drugs.com (2022) |
| Digoxin | 2.820 | 7.140 | 0.849 | 0.610 | 0.940 | metrumresearchgroup/PBPK_PC (2020) |
| Fluoxetine | 21.000 | 0.050 | 0.970 | 0.055 | 0.960 | U.S. Food and Drug Administration (1987)  Sager et al. (2014)  Jeong et al. (2021) |
| Metoprolol | 195.000 | 0.000 | 1.450 | 0.879 | 1.127 | metrumresearchgroup/PBPK_PC (2020) |
| Midazolam | 32.400 | 0.000 | 3.040 | 0.032 | 0.664 | DrugBank Online (n.d.c)  metrumresearchgroup/PBPK_PC (2020) |
| Nevirapine | 1.260 | 0.070 | 0.670 | 0.400 | 1.040 | metrumresearchgroup/PBPK_PC (2020) |
| Ofloxacin | 2.760 | 8.920 | 0.849 | 0.770 | 0.920 | metrumresearchgroup/PBPK_PC (2020) |
| Paracetamol | 19.700 | 0.000 | 0.849 | 0.880 | 1.600 | Forrest et al. (1982)  Villiger et al. (2016) |
| Pioglitazone | 5.000 | 0.000 | 0.849 | 0.015 | 1.000 | Xiao et al. (1982)  U.S. Food and Drug Administration (1999)  Budde et al. (2003) |
| Rifampicin | 7.000 | 1.500 | 0.849 | 0.340 | 0.670 | U.S. Food and Drug Administration (2013)  Rasool et al. (2019) |
| *S*-Warfarin | 0.209 | 0.000 | 0.849 | 0.009 | 0.590 | Holford (1986)  Ichihara et al. (2015)  Wang et al. (2021) |
| Telmisartan | 48.000 | 0.000 | 0.680 | 0.005 | 0.780 | Stangier et al. (2000)  Bae et al. (2018) |
| Theophylline | 2.340 | 0.382 | 0.316 | 0.280 | 0.850 | DrugBank Online (n.d.d)  Mitenko and Ogilvie (1974)  Ebden et al. (1986)  Ginsberg et al. (2004)  Britz et al. (2019) |
| Thiopental | 13.800 | 0.000 | 0.849 | 0.130 | 1.000 | metrumresearchgroup/PBPK_PC (2020) |
| Voriconazole | 7.649 | 0.096 | 0.849 | 0.420 | 1.000 | Yanni et al. (2010)  Zane and Thakker (2014)  metrumresearchgroup/PBPK_PC (2020) |

*CL_he_****_p_***, hepatic clearance; *CL_ki_*, renal clearance; *K_a_*, absorption rate constant; *f_u_*, drug unbound fraction; *BP*, blood to plasma ratio.

**References**

Bae, S.H., Park, W.S., Han, S., Park, G.J., Lee, J., Hong, T., et al. (2018). Physiologically-based pharmacokinetic predictions of intestinal BCRP-mediated drug interactions of rosuvastatin in Koreans. *Korean. J. Physiol. Pharmacol*. 22, 321-329. doi: 10.4196/kjpp.2018.22.3.321

Bower, S., and Hull, C.J. (1982). Comparative pharmacokinetics of fentanyl and alfentanil. *Br. J. Anaesth*. 54, 871-877. doi: 10.1093/bja/54.8.871

Britz, H., Hanke, N., Volz, A.K., Spigset, O., Schwab, M., Eissing, T., et al. (2019). Physiologically-based pharmacokinetic models for CYP1A2 drug-drug interaction prediction: A modeling network of fluvoxamine, theophylline, caffeine, rifampicin, and midazolam. *CPT: Pharmacomet. Syst. Pharmacol*. 8, 296-307. doi: 10.1002/psp4.12397

Budde, K., Neumayer, H.H., Fritsche, L., Sulowicz, W., Stompôr, T., and Eckland, D. (2003). The pharmacokinetics of pioglitazone in patients with impaired renal function. *Br. J. Clin. Pharmacol*. 55, 368-374. doi: 10.1046/j.1365-2125.2003.01785.x

DrugBank Online. (n.d.a). Amlodipine: Uses, interaction, mechanism of action. https://go.drugbank.com/drugs/DB00381 [Accessed January 10, 2021].

DrugBank Online. (n.d.b). Cyclosporin: Uses, interaction, mechanism of action. https://go.drugbank.com/drugs/DB00091 [Accessed January 10, 2021].

DrugBank Online. (n.d.c). Midazolam: Uses, interaction, mechanism of action. https://go.drugbank.com/drugs/DB00683 [Accessed January 10, 2021].

DrugBank Online. (n.d.d). Theophylline: Uses, interaction, mechanism of action. https://go.drugbank.com/drugs/DB00277 [Accessed January 10, 2021].

Drugs.com. (2022). Cyclosporine - FDA prescribing information, side effects and uses. https://www.drugs.com/pro/cyclosporine.html [Accessed January 10, 2021].

Ebden, P., Banks, J., Peel, T., Buss, D.C., Routledge, P.A., and Spragg, B.P. (1986). The disposition of theophylline in blood in chronic obstructive lung disease. *Ther. Drug Monit*. 8, 424-426. doi: 10.1097/00007691-198612000-00008

Forrest, J.A.H., Clements, J.A., and Prescott, L.F. (1982). Clinical pharmacokinetics of paracetamol. *Clin. Pharmacokinet*. 7, 93-107. doi: 10.2165/00003088-198207020-00001

Ghoneim, A.M., and Mansour, S.M. (2020). The effect of liver and kidney disease on the pharmacokinetics of clozapine and sildenafil: A physiologically based pharmacokinetic modeling. *Drug. Des. Devel. Ther*. 14, 1469-1479. doi: 10.2147/DDDT.S246229

Ginsberg, G., Hattis, D., Russ, A., and Sinawane, B. (2004). Physiologically based pharmacokinetic (PBPK) modeling of caffeine and theophylline in neonates and adults: Implications for assessing children’s risks from environmental agents. *J. Toxicol. Environ. Health Part A*. 67, 297-329. doi: 10.1080/15287390490273550

Holford, N.H.G. (1986). Clinical pharmacokinetics and pharmacodynamics of warfarin. *Clin. Pharmacokinet*. 11, 483-504. doi: 10.2165/00003088-198611060-00005

Ichihara, N., Ishigami, T., and Umemura, S. (2015). Effect of impaired renal function on the maintenance dose of warfarin in Japanese patients. *J. Cardiol*. 65, 178-184. doi: 10.1016/j.jjcc.2014.08.008

Jeong, H., Chae, Y., Lee, S., Kang, W., Yun, H., and Shin, K. (2021). Prediction of fluoxetine and norfluoxetine pharmacokinetic profiles using physiologically based pharmacokinetics modeling. *J. Clin. Pharmacol*. 61, 1505-1513. doi: 10.1002/jcph.1927

Lee, J., Kim, M., Jeong, H., and Shin, K. (2021). Physiologically-based pharmacokinetics model for clozapine in Korean patients with schizophrenia. *Transl. Clin. Pharmacol*. 29, 33-44. doi: 10.12793/tcp.2021.29.e3

Lee, Y., Ryu, S., Kim, J., and Shin, H. (2007). Factors influencing the cyclosporine clearance in allergenic hematopoietic cell transplant recipients. *J. Kor. Soc. Health-Syst. Pharm*. 24, 312-317. doi: 10.32429/jkshp.2007.24.4.002

metrumresearchgroup/PBPK_PC. (2020). PBPK_PC. https://github.com/metrumresearchgroup/PBPK_PC/tree/master/model [Accessed January 10, 2021].

Mitenko, P.A., and Ogilvie, R.I. (1974). Bioavailability and efficacy of a sustained-release theophylline tablet. *Clin. Pharmacol. Ther*. 16, 720-726. doi: 10.1002/cpt1974164720

Mukherjee, D., Zha, J., Menon, R.M., and Shebley, M. (2018). Guiding dose adjustment od amlodipine after co-administration with ritonavir containing regimens using a physiologically-based pharmacokinetic/pharmacodynamic model. *J. Pharmacokinet. Pharmacodyn*. 45, 443-456. doi: 10.1007/s10928-018-9574-0

Rasool, M.F., Khalid, S., Majeed, A., Saeed, H., Imran, I., Mohany, M., et al. (2019). Development and evaluation of physiologically based pharmacokinetic drug-disease models for predicting rifampicin exposure in tuberculosis and cirrhosis populations. *Pharmaceutics*. 11:578. doi: 10.3390/pharmaceutics11110578

Sager, J.E., Lutz, J.D., Foti, R.S., Davis, C., Kunze, K.L., and Isoherranen, N. (2014). Fluoxetine- and norfluoxetine-mediated complex drug-drug interactions: In vitro to in vivo correlation of effects on CYP2D6, CYP2C19, and CYP3A4. *Clin. Pharmacol. Ther*. 95, 653-662. doi: 10.1038/clpt.2014.50

Schaber, G., Stevens, I., Gaertner, H.J., Diet, K., and Breyeer-Pfaff, U. (1998). Pharmacokinetics of clozapine and its metabolites in psychiatric patients: Plasma protein binding and renal clearance. *Br. J. Clin. Pharmacol*. 46, 453-459. doi: 10.1046/j.1365-2125.1998.00822.x

Stangier, J., Su, C., and Roth, W. (2000). Pharmacokinetics of orally and intravenously administered telmisartan in healthy young and elderly volunteers and in hypertensive patients. *J. Int. Med. Res*. 28, 149-167. doi: 10.1177/147323000002800401

U.S. Food and Drug Administration. (1987). Prozac (fluoxetine hydrochloride) capsules label. https://www.accessdata.fda.gov/drugsatfda_docs/label/2011/018936s091lbl.pdf [Accessed January 10, 2021].

U.S. Food and Drug Administration. (1999). ACTOS^TM^ (Pioglitazone Hydrochloride) Tablet NDA No. 21-073. https://www.accessdata.fda.gov/drugsatfda_docs/label/1999/21073lbl.pdf [Accessed January 10, 2021].

U.S. Food and Drug Administration. (2013). RIFADIN^®^ (rifampin capsules USP) and RIFADIN^®^ IV (rifampin for injection USP). https://www.accessdata.fda.gov/drugsatfda_docs/label/2013/050420s075,050627s014lbl.pdf [Accessed January 10, 2021].

Villiger, A., Stillhart, C., Parrott, N., and Kuentz, M. (2016). Using physiologically based pharmacokinetic (PBPK) modelling to gain insights into the effect of physiological factors on oral absorption in paediatric populations. *AAPS. J*. 18, 933-947. doi: 10.1208/s12248-016-9896-z

Wang, Z., Xiang, X., Liu, S., Tang, Z., Sun, H., Parvez, M., et al. (2021). A physiologically based pharmacokinetic/pharmacodynamic modeling approach for drug-drug interaction evaluation of warfarin enantiomers with sorafenib. *Drug. Metab. Pharmacokinet*. 39:100362. doi: 10.1016/j.dmpk.2020.10.001

Xiao, Q., Tang, L., Xu, R., Qian, W., and Yang, J. (2015). Physiologically based pharmacokinetics model predicts the lack of inhibition by repaglinide on the metabolism of pioglitazone. *Br. Biopharm. Drug. Dispos*. 36, 603-612. doi: 10.1002/bdd.1987

Yanni, S.B., Annaert, P.P., Augustijns, P., Ibrahim, J.G., Benjamin Jr, D.K., and Thakker, D.R. (2010). In vitro hepatic metabolism explains higher clearance of voriconazole in children versus adults: Role of CYP2C19 and Flavin-containing monooxygenase 3. *Drug. Metab. Dispos*. 38, 25-31. doi: 10.1124/dmd.109.029769

Yoon, S., Yi, S., Rhee, S.J., Lee, H.A., Kim, Y., Yu, K.S., et al. (2019). Development of a physiologically-based pharmacokinetic model for cyclosporine in Asian children with renal impairment. *Transl. Clin. Pharmacol*. 27, 107-114. doi: 10.12793/tcp.2019.27.3.107

Zane, N.R., and Thakker, D.R. (2014). A physiologically based pharmacokinetic model for voriconazole disposition predicts intestinal first-pass metabolism in children. *Clin. Pharmacokinet*. 53, 1171-1182. doi: 10.1007/s40262-014-0181-y

Table S4. Input parameters of lumped central compartment (LCEN) in lumped models for 20 compounds

| Parameter (unit)  Compound | *V_LCEN_* (L) | *Q_LCEN_* (L/h) | *K_Plu_* |
| --- | --- | --- | --- |
| Alfentanil | 6.100 | 390.000 | 1.231 |
| Amlodipine | 6.100 | 390.000 | 13.898 |
| Artemether | 4.320 | 354.000 | 1.226 |
| Caffeine | 4.320 | 354.000 | 1.208 |
| Clozapine | 6.100 | 390.000 | 12.943 |
| Cyclosporine A | 6.100 | 390.000 | 3.618 |
| Digoxin | 6.100 | 390.000 | 1.282 |
| Fluoxetine | 4.320 | 354.000 | 1.928 |
| Metoprolol | 4.320 | 354.000 | 1.341 |
| Midazolam | 4.320 | 354.000 | 1.215 |
| Nevirapine | 4.320 | 354.000 | 1.197 |
| Ofloxacin | 6.100 | 390.000 | 1.300 |
| Paracetamol | 6.100 | 390.000 | 1.353 |
| Pioglitazone | 6.100 | 390.000 | 1.028 |
| Rifampicin | 4.320 | 354.000 | 1.932 |
| *S*-Warfarin | 6.100 | 390.000 | 1.028 |
| Telmisartan | 4.320 | 354.000 | 0.991 |
| Theophylline | 4.320 | 354.000 | 1.092 |
| Thiopental | 6.100 | 390.000 | 1.309 |
| Voriconazole | 6.100 | 390.000 | 1.333 |

*V_LCEN_*, volume in the LCEN; *Q_LCEN_*, blood flow in the LCEN; *K_Plu_*, lung to plasma partition coefficient.

Table S5. Input parameters of non-eliminating tissues compartment (NET) in lumped models for 20 compounds

| Parameter (unit)  Compound | *V_NET_* (L) | *Q_NET_* (L/h) | *K_PNET_* |
| --- | --- | --- | --- |
| Alfentanil | 59.530 | 216.450 | 1.776 |
| Amlodipine | 62.530 | 216.450 | 14.342 |
| Artemether | 52.725 | 198.240 | 4.518 |
| Caffeine | 52.725 | 198.240 | 0.417 |
| Clozapine | 59.530 | 216.450 | 8.875 |
| Cyclosporine A | 59.530 | 216.450 | 1.655 |
| Digoxin | 62.530 | 216.450 | 0.884 |
| Fluoxetine | 52.725 | 198.240 | 6.358 |
| Metoprolol | 52.725 | 198.240 | 2.215 |
| Midazolam | 52.725 | 198.240 | 3.471 |
| Nevirapine | 52.725 | 198.240 | 1.318 |
| Ofloxacin | 62.530 | 216.450 | 0.515 |
| Paracetamol | 62.530 | 216.450 | 0.903 |
| Pioglitazone | 62.530 | 216.450 | 0.097 |
| Rifampicin | 52.725 | 198.240 | 1.337 |
| *S*-Warfarin | 62.530 | 216.450 | 0.099 |
| Telmisartan | 52.725 | 198.240 | 0.098 |
| Theophylline | 52.725 | 198.240 | 0.428 |
| Thiopental | 62.530 | 216.450 | 4.296 |
| Voriconazole | 63.640 | 220.350 | 5.912 |

*V_NET_*, volume in the NET; *Q_NET_*, blood flow in the NET; *K_PNET_*, tissue to plasma partition coefficient in the NET.

Table S6. Input parameters for 20 compounds in the compartment model

| Parameter (unit)  Compound | *CL* (L/h) | *V_c_* (L) | *V_p_* (L) | *Q* (L/h) | *K_a_* (1/h) | Source |
| --- | --- | --- | --- | --- | --- | --- |
| Alfentanil | 14.600 | 145.000 | - | - | 17.700 | Pérus et al. (2012) |
| Amlodipine | 18.595 | 1242.380 | - | - | 0.640 | Rohatagi et al. (2008) |
| Artemether | 986.182 | 1410.545 | - | - | 0.392 | Tarning et al. (2014) |
| Caffeine | 5.657 | 39.429 | - | - | 0.642 | Seng et al. (2009) |
| Clozapine | 21.900 | 526.000 | - | - | 1.300 | Li et al. (2012) |
| Cyclosporine A | 32.126 | 149.919 | - | - | 1.280 | Wu et al. (2005) |
| Digoxin | 16.209 | 180.101 | 576.325 | 57.632 | 3.150 | Comets et al. (2007) |
| Fluoxetine | 12.465 | 19.070 | - | - | 0.016 | Tanoshima et al. (2012) |
| Metoprolol | 169.286 | 41.186 | - | - | 0.200 | Eugene et al. (2012) |
| Midazolam | 53.743 | 114.000 | 125.143 | 29.743 | 3.770 | Zhou et al. (2014) |
| Nevirapine | 0.917 | 51.771 | 54.086 | 16.543 | 0.677 | Ibarra et al. (2014) |
| Ofloxacin | 9.667 | 61.028 | 45.936 | 29.721 | 0.849 | Preston et al. (1998) |
| Paracetamol | 15.709 | 50.730 | 27.999 | 3.271 | 0.849 | Mian et al. (2019) |
| Pioglitazone | 2.576 | 22.070 | 26.264 | 0.614 | 2.080 | Kadam et al. (2013) |
| Rifampicin | 9.778 | 29.335 | - | - | 2.150 | Seng et al. (2015) |
| *S*-Warfarin | 0.135 | 13.750 | - | - | 1.660 | Lane et al. (2011) |
| Telmisartan | 58.800 | 192.000 | 996.000 | 79.700 | 0.330 | Tatami et al. (2003) |
| Theophylline | 3.234 | 19.200 | - | - | 0.077 | Tanigawara et al. (1995) |
| Thiopental | 8.353 | 151.214 | 140.413 | 1.966 | 0.849 | Russo et al. (1997) |
| Voriconazole | 6.424 | 82.386 | 107.414 | 16.164 | 100.000 | Friberg et al. (2012) |
| Inter-individual variability | 0.100 | 0.100 | 0.100^*^ | 0.100^*^ | 0.100 | - |
| Residual variability | 0.26 | | | | | - |

*CL*, clearance; *V_c_*, central volume of distribution; *V_p_*, peripheral volume of distribution; *Q,* intercompartmental clearance; *K_a_*, absorption rate constant. ^*^Inter-individual variability for 2-compartment model.

**References**

Comets, E., Verstuyft, C., Lavielle, M., Jaillon, P., Becquemont, L., and Mentré, F. (2007). Modelling the influence of MDR1 polymorphism on digoxin pharmacokinetic parameters. *Eur. J. Clin. Pharmacol*. 63, 437-449. doi: 10.1007/s00228-007-0269-5

Eugene, A.R. (2016). Metoprolol dose equivalence in adult men and women based on gender differences: Pharmacokinetic modeling and simulations. *Med. Sci*. 4:18. doi: 10.3390/medsci4040018

Friberg, L.E., Ravva, P., Karlsson, M.O., and Liu, P. (2012). Integrated population pharmacokinetic analysis of voriconazole in children, adolescents, and adults. *Antimicrob. Agents Chemother*. 56, 3032-3042. doi: 10.1128/AAC.05761-11

Ibarra, M., Vázquez, M., and Fagiolino, P. (2014). Population pharmacokinetic model to analyze nevirapine multiple-peaks profile after a single oral dose. *J. Pharmacokinet. Pharmacodyn*. 41, 363-373. doi: 10.1007/s10928-014-9371-3

Kadam, R., Bourne, D., Kompella, U., and Aquilante, C. (2013). Effect of cytochrome P450 2C8*3 on the population pharmacokinetics of pioglitazone in healthy caucasian volunteers. *Biol. Pharm. Bull*. 36, 245-251. doi: 10.1248/bpb.b12-00657

Lane, S., Al-Zubiedi, S., Hatch, E., Matthews, I., Jorgensen, A.L., Deloukas, P., et al. (2012). The population pharmacokinetics of R- and S-warfarin: Effect of genetic and clinical factors. *Br. J. Clin. Pharmacol*. 73, 66-76. doi: 10.1111/j.1365-2125.2011.04051.x

Li, L.J., Shang, D.W., Li, W.B., Guo, W., Wang, X.P., Ren, Y.P., et al. (2012). Population pharmacokinetics of clozapine and its primary metabolite norclozapine in Chinese patients with schizophrenia. *Acta Pharmacol. Sin*. 33, 1409-1416. doi: 10.1038/aps.2012.71

Mian, P., van Esdonk, M.J., Olkkola, K.T., de Winter, B., Liukas, A., Spriet, I., et al. (2019). Population pharmacokinetic modelling of intravenous paracetamol in fit older people displays extensive unexplained variability. *Br. J. Clin. Pharmacol*. 85, 126-135. doi: 10.1111/bcp.13770

Pérus, O., Marsot, A., Ramain, E., Dahman, M., Paci, A., Raucoules-Aimé, M., et al. (2012). Performance of alfentanil target-controlled infusion in normal and morbidly obese female patients. *Br. J. Anaesth*. 109, 551-560. doi: 10.1093/bja/aes211

Preston, S.L., Drusano, G.L., Berman, A.L., Fowler, C.L., Chow, A.T., Dornseif, B., et al. (1998). Levofloxacin population pharmacokinetics and creation of a demographic model for prediction of individual drug clearance in patients with serious community-acquired infection. *Antimicrob. Agents Chemother*. 42, 1098-1104. doi: 10.1128/AAC.42.5.1098

Rohatagi, S., Carrothers, T.J., Kshirsagar, S., Khariton, T., Lee, J., and Salazar, D. (2008). Evaluation of population pharmacokinetics and exposure-response relationship with coadministration of amlodipine besylate and olmesartan medoxomil. *J. Clin. Pharmacol*. 48, 823-836. doi: 10.1177/0091270008317847

Russo, H., Simon, N., Duboin, M.P., and Urien, S. (1997). Population pharmacokinetics of high-dose thiopental in patients with cerebral injuries. *Clin. Pharmcol. Ther*. 62, 15-20. doi: 10.1016/S0009-9236(97)90147-8

Seng, K.Y., Fun, C.Y., Law, Y.L., Lim, W.M., Fan, W., and Lim, C.L. (2009). Population pharmacokinetics of caffeine in healthy male adults using mixed-effects models. *J. Clin. Pharm. Ther*. 34, 103-114. doi: 10.1111/j.1365-2710.2008.00976.x

Seng, K.Y., Hee, K.H., Soon, G.H., Chew, N., Khoo, S.H., and Lee, L.S.U. (2015). Population pharmacokinetics of rifampicin and 25-deacetyl-rifampicin in healthy Asian adults. *J. Antimicrob. Chemother*. 70, 3298-3306. doi: 10.1093/jac/dkv268

Tanigawara, Y., Komada, F., Shimizu, T., Iwakawa, S., Iwai, T., Maekawa, H., et al. (1995). Population pharmacokinetics of theophylline. III. Premarketing study for a once-daily administered preparation. *Biol. Pharm. Bull*. 18, 1590-1598. doi: 10.1248/bpb.18.1590

Tanoshima, R., Bournissen, F.G., Tanigawara, Y., Kristensen, J.H., Taddio, A., Ilett, K.F., et al. (2014). Population PK modelling and simulation based on fluoxetine and norfluoxetine concentrations in milk: A milk concentration-based prediction model. *Br. J. Clin. Pharmacol*. 78, 918-928. doi: 10.1111/bcp.12409

Tarning, J., Kloprogge, F., Piola, P., Dhorda, M., Muwanga, S., Turyakira, E., et al. (2012). Population pharmacokinetics of artemether and dihydroartemisinin in pregnant women with uncomplicated Plasmodium falciparum malaria in Uganda. *Malar. J*. 11:293. doi: 10.1186/1475-2875-11-293

Tatami, S., Sarashina, A., Yamamura, N., Igarashi, T., and Tanigawara, Y. (2003). Population pharmacokinetics of an angiotensin II receptor antagonist, telmisartan, in healthy volunteers and hypertensive patients. *Drug. Metab. Pharmacokinet*. 18, 203-211. doi: 10.2133/dmpk.18.203

Wu, K.H., Cui, Y.M., Guo, J.F., Zhou, Y., Zhai, S.D., Cui, F.D., et al. (2005). Population pharmacokinetics of cyclosporine in clinical renal transplant patients. *Drug Metab. Dispos*. 33, 1268-1275. doi: 10.1124/dmd.105.004358

Zhou, D., Lu, Z., Sunzel, M., Xu, H., and Al-Huniti, N. (2014). Population pharmacokinetic modelling to assess clinical drug-drug interaction between AZD7325 and midazolam. *J. Clin. Pharm. Ther*. 39, 404-410. doi: 10.1111/jcpt.12152

Table S7. Comparison of *V_c_* parameters in PBPK, lumped, and compartment models for 20 compounds

| Parameter (unit) | *V_c_* (L/kg) | | |
| --- | --- | --- | --- |
| Model  Compound | PBPK model  (2-fold range) | Lumped model  (2-fold range) | Compartment model  (2-fold range) |
| Alfentanil | 1.000 (0.500-2.000) | 1.000 (0.500-2.000) | 2.071 (1.036-4.143) |
| Amlodipine | 1.000 (0.500-2.000) | 1.000 (0.500-2.000) | 17.019 (8.509-34.038) |
| Artemether | 1.000 (0.500-2.000) | 1.000 (0.500-2.000) | 23.509 (11.755-47.018) |
| Caffeine | 1.000 (0.500-2.000) | 1.000 (0.500-2.000) | 0.657 (0.329-1.314) |
| Clozapine | 1.000 (0.500-2.000) | 1.000 (0.500-2.000) | 7.514 (3.757-15.029) |
| Cyclosporine A | 1.000 (0.500-2.000) | 1.000 (0.500-2.000) | 2.142 (1.071-4.283) |
| Digoxin | 1.000 (0.500-2.000) | 1.000 (0.500-2.000) | 2.467 (1.234-4.934) |
| Fluoxetine | 1.000 (0.500-2.000) | 1.000 (0.500-2.000) | 0.318 (0.159-0.636) |
| Metoprolol | 1.000 (0.500-2.000) | 1.000 (0.500-2.000) | 0.686 (0.343-1.373) |
| Midazolam | 1.000 (0.500-2.000) | 1.000 (0.500-2.000) | 1.900 (0.950-3.800) |
| Nevirapine | 1.000 (0.500-2.000) | 1.000 (0.500-2.000) | 0.863 (0.431-1.726) |
| Ofloxacin | 1.000 (0.500-2.000) | 1.000 (0.500-2.000) | 0.836 (0.418-1.672) |
| Paracetamol | 1.000 (0.500-2.000) | 1.000 (0.500-2.000) | 0.695 (0.347-1.390) |
| Pioglitazone | 1.000 (0.500-2.000) | 1.000 (0.500-2.000) | 0.302 (0.151-0.605) |
| Rifampicin | 1.000 (0.500-2.000) | 1.000 (0.500-2.000) | 0.489 (0.244-0.978) |
| *S*-Warfarin | 1.000 (0.500-2.000) | 1.000 (0.500-2.000) | 0.188 (0.094-0.377) |
| Telmisartan | 1.000 (0.500-2.000) | 1.000 (0.500-2.000) | 3.200 (1.600-6.400) |
| Theophylline | 1.000 (0.500-2.000) | 1.000 (0.500-2.000) | 0.320 (0.160-0.640) |
| Thiopental | 1.000 (0.500-2.000) | 1.000 (0.500-2.000) | 2.071 (1.036-4.143) |
| Voriconazole | 1.000 (0.500-2.000) | 1.000 (0.500-2.000) | 1.129 (0.564-2.257) |

*V_c_*, central volume of distribution.

Table S8. Comparison of *V_p_* parameters in PBPK, lumped, and compartment models for 20 compounds

| Parameter (unit) | *V_p_* (L/kg) | | |
| --- | --- | --- | --- |
| Model  Compound | PBPK model  (2-fold range) | Lumped model  (2-fold range) | Compartment model  (2-fold range) |
| Alfentanil | 0.252 (0.126-0.503) | 0.452 (0.226-0.903) | - |
| Amlodipine | 2.048 (1.024-4.095) | 1.749 (0.874-3.498) | - |
| Artemether | 0.567 (0.284-1.134) | 0.828 (0.414-1.655) | - |
| Caffeine | 0.052 (0.026-0.105) | 0.074 (0.037-0.149) | - |
| Clozapine | 1.258 (0.629-2.516) | 1.334 (0.667-2.669) | - |
| Cyclosporine A | 0.235 (0.117-0.469) | 0.332 (0.166-0.664) | - |
| Digoxin | 0.126 (0.063-0.253) | 0.172 (0.086-0.344) | 7.895 (3.947-15.790) |
| Fluoxetine | 0.798 (0.399-1.596) | 1.217 (0.608-2.433) | - |
| Metoprolol | 0.278 (0.139-0.556) | 0.593 (0.297-1.187) | - |
| Midazolam | 0.436 (0.218-0.872) | 0.748 (0.374-1.495) | 2.086 (1.043-4.171) |
| Nevirapine | 0.165 (0.083-0.331) | 0.250 (0.125-0.501) | 0.901 (0.451-1.803) |
| Ofloxacin | 0.074 (0.037-0.147) | 0.085 (0.043-0.170) | 0.629 (0.315-1.259) |
| Paracetamol | 0.129 (0.064-0.258) | 0.128 (0.064-0.0256) | 0.384 (0.192-0.767) |
| Pioglitazone | 0.014 (0.007-0.028) | 0.016 (0.008-0.031) | 0.360 (0.180-0.720) |
| Rifampicin | 0.168 (0.084-0.336) | 0.140 (0.070-0.279) | - |
| *S*-Warfarin | 0.014 (0.007-0.028) | 0.016 (0.008-0.032) | - |
| Telmisartan | 0.012 (0.006-0.025) | 0.016 (0.008-0.032) | 16.600 (8.300-33.200) |
| Theophylline | 0.054 (0.027-0.107) | 0.056 (0.028-0.112) | - |
| Thiopental | 0.613 (0.307-1.227) | 0.938 (0.469-1.876) | 1.923 (0.962-3.847) |
| Voriconazole | 0.859 (0.430-1.718) | 0.824 (0.412-1.649) | 1.471 (0.736-2.943) |

*V_p_*, peripheral volume of distribution.

TABLE S9. Comparison of C_max_ parameters of central compartment in PBPK, lumped, and compartment models for 20 compounds

| Model | PBPK model | Lumped model | Compartment model |
| --- | --- | --- | --- |
| Tissue, compartment | Lungs, arterial blood, venous blood | Lumped central compartment (LCEN) | Central compartment |
| Parameter (unit)  Compound | Average of tissue C_max_ (mg/L)  (2-fold range) | C_max_ at LCEN (mg/L)  (2-fold range) | C_max_ at central compartment (mg/L)  (2-fold range) |
| Alfentanil | 0.044 (0.022-0.089) | 0.034 (0.017-0.068) | 0.053 (0.026-0.106) |
| Amlodipine | 0.316 (0.158-0.633) | 0.017 (0.008-0.034) | 0.007 (0.004-0.015) |
| Artemether | 1.471 (0.736-2.942) | 2.462 (1.231-4.923) | 0.030 (0.015-0.061) |
| Caffeine | 0.216 (0.108-0.432) | 0.186 (0.093-0.371) | 0.082 (0.041-0.165) |
| Clozapine | 9.670 (4.835-19.340) | 0.682 (0.341-1.364) | 0.508 (0.254-1.016) |
| Cyclosporine A | 4.263 (2.132-8.526) | 2.231 (1.116-4.462) | 2.549 (1.275-5.098) |
| Digoxin | 0.005 (0.002-0.010) | 0.005 (0.003-0.010) | 0.002 (0.001-0.004) |
| Fluoxetine | 0.536 (0.268-1.072) | 0.939 (0.470-1.879) | 0.069 (0.035-0.138) |
| Metoprolol | 0.998 (0.499-1.996) | 0.920 (0.460-1.839) | 0.100 (0.050-0.199) |
| Midazolam | 0.069 (0.034-0.137) | 0.108 (0.054-0.216) | 0.030 (0.015-0.059) |
| Nevirapine | 4.682 (2.341-9.365) | 3.481 (1.740-6.962) | 2.144 (1.072-4.288) |
| Ofloxacin | 6.720 (3.360-13.440) | 7.181 (3.590-14.362) | 3.920 (1.960-7.839) |
| Paracetamol | 5.192 (2.596-10.385) | 6.122 (3.061-12.244) | 10.299 (5.149-20.597) |
| Pioglitazone | 0.442 (0.221-0.884) | 0.586 (0.293-1.172) | 0.556 (0.278-1.112) |
| Rifampicin | 36.014 (18.007-72.029) | 13.986 (6.993-27.971) | 14.525 (7.262-29.049) |
| *S*-Warfarin | 0.368 (0.184-0.735) | 0.456 (0.228-0.913) | 0.353 (0.176-0.705) |
| Telmisartan | 3.673 (1.836-7.346) | 4.516 (2.258-9.032) | 0.051 (0.025-0.101) |
| Theophylline | 9.001 (4.501-18.003) | 7.890 (3.945-15.780) | 2.464 (1.232-4.929) |
| Thiopental | 90.292 (45.146-180.585) | 69.300 (34.650-138.601) | 128.815 (64.408-257.630) |
| Voriconazole | 1.573 (0.787-3.147) | 1.138 (0.569-2.276) | 4.737 (2.369-9.474) |

C_max_, maximum blood concentration.

TABLE S10. Comparison of C_max_ parameters of peripheral compartment in PBPK, lumped, and compartment models for 20 compounds

| Model | PBPK model | Lumped model | Compartment model |
| --- | --- | --- | --- |
| Tissue, compartment | Adipose, bone, brain, muscle, skin, heart, rest of body | Non-eliminating tissues compartment (NET) | Peripheral compartment |
| Parameter (unit)  Compound | Average of tissue C_max_ (mg/L)  (2-fold range) | C_max_ at NET (mg/L)  (2-fold range) | C_max_ at peripheral compartment (mg/L)  (2-fold range) |
| Alfentanil | 0.190 (0.095-0.379) | 0.088 (0.044-0.177) | - |
| Amlodipine | 0.246 (0.123-0.493) | 0.164 (0.082-0.328) | - |
| Artemether | 11.777 (5.888-23.554) | 2.613 (1.306-5.225) | - |
| Caffeine | 0.139 (0.070-0.278) | 0.073 (0.036-0.146) | - |
| Clozapine | 7.814 (3.907-15.628) | 3.501 (1.751-7.003) | - |
| Cyclosporine A | 3.244 (1.622-6.487) | 2.226 (1.113-4.452) | - |
| Digoxin | 0.007 (0.003-0.013) | 0.005 (0.002-0.010) | 0.0005 (0.0002-0.0010) |
| Fluoxetine | 3.552 (1.776-7.104) | 0.828 (0.414-1.655) | - |
| Metoprolol | 4.746 (2.373-9.491) | 1.096 (0.548-2.193) | - |
| Midazolam | 0.494 (0.247-0.988) | 0.123 (0.061-0.245) | 0.009 (0.004-0.017) |
| Nevirapine | 8.201 (4.100-16.402) | 3.095 (1.547-6.190) | 1.723 (0.861-3.445) |
| Ofloxacin | 4.595 (2.297-9.189) | 3.999 (1.999-7.997) | 3.275 (1.637-6.550) |
| Paracetamol | 3.375 (1.688-6.750) | 3.426 (1.713-6.852) | 3.469 (1.734-6.938) |
| Pioglitazone | 0.065 (0.032-0.130) | 0.057 (0.029-0.114) | 0.076 (0.038-0.153) |
| Rifampicin | 26.065 (13.032-52.130) | 13.319 (6.659-26.637) | - |
| *S*-Warfarin | 0.088 (0.044-0.175) | 0.076 (0.038-0.153) | - |
| Telmisartan | 0.799 (0.399-1.598) | 0.565 (0.282-1.129) | 0.016 (0.008-0.033) |
| Theophylline | 5.318 (2.659-10.637) | 3.634 (1.817-7.269) | - |
| Thiopental | 523.698 (261.849-1047.397) | 258.663 (129.331-517.326) | 21.709 (10.855-43.419) |
| Voriconazole | 6.466 (3.233-12.932) | 4.845 (2.422-9.689) | 1.492 (0.746-2.985) |

C_max_, maximum blood concentration.

Table S11. Comparison of AUC parameter of voriconazole in PBPK, lumped, and compartment models

| Model | | | PBPK model | Lumped model | Compartment model |
| --- | --- | --- | --- | --- | --- |
| Compartment in compartment model | Compartment in lumped model | Parameter (unit)  Tissue | Tissue AUC_last_  (mg•h/L)  (2-fold range) | AUC_last_ at LCEN/NET  (mg•h/L)  (2-fold range) | AUC_last_ at central/peripheral compartment (mg•h/L)  (2-fold range) |
| Central compartment | Lumped central compartment (LCEN) | Original lungs | 38.350  (19.175-76.701) | 46.360  (23.180-92.720) | 63.400  (31.700-126.802) |
|  |  | Original arterial blood | 46.205  (23.102-92.410) |  |  |
|  |  | Original venous blood | 46.205  (23.103-92.411) |  |  |
| Peripheral compartment | Non-eliminating tissues compartment (NET) | Original adipose | 447.884  (223.942-895.768) | 273.008  (136.504-546.016) | 63.054  (31.527-126.109) |
|  |  | Original bone | 363.089  (181.545-726.179) |  |  |
|  |  | Original brain | 339.651  (169.826-679.303) |  |  |
|  |  | Original muscle | 135.747  (67.874-271.494) |  |  |
|  |  | Original heart | 90.090  (45.045-180.180) |  |  |
|  |  | Original rest of body | 184.886  (92.443-369.773) |  |  |
| Remaining tissue | | Original kidneys | 133.917  (66.959-267.835) | 134.365  (67.183-268.730) | - |
|  |  | Original spleen | 136.753  (68.376-273.505) | 137.205  (68.602-274.410) | - |
|  |  | Original gut | 309.982  (154.991-619.964) | 310.859  (155.429-621.717) | - |
|  |  | Original liver | 216.642  (108.321-433.285) | 217.267  (108.633-434.533) | - |

AUC, area under the concentration-time curve.
